# Is There a Conjunction Fallacy in Legal Probabilistic Decision Making?

**DOI:** 10.3389/fpsyg.2018.00391

**Published:** 2018-04-05

**Authors:** Bartosz W. Wojciechowski, Emmanuel M. Pothos

**Affiliations:** ^1^Department of Clinical and Forensic Psychology, Institute of Psychology, University of Silesia of Katowice, Katowice, Poland; ^2^Department of Psychology, City, University of London, London, United Kingdom

**Keywords:** conjunction fallacy, legal decision making, quantum cognition, quantum probability theory, legal psychology

## Abstract

Classical probability theory (CPT) has represented the rational standard for decision making in human cognition. Even though CPT has provided many descriptively excellent decision models, there have also been some empirical results persistently problematic for CPT accounts. The tension between the normative prescription of CPT and human behavior is particularly acute in cases where we have higher expectations for rational decisions. One such case concerns legal decision making from legal experts, such as attorneys and prosecutors and, more so, judges. In the present research we explore one of the most influential CPT decision fallacies, the conjunction fallacy (CF), in a legal decision making task, involving assessing evidence that the same suspect had committed two separate crimes. The information for the two crimes was presented consecutively. Each participant was asked to provide individual ratings for the two crimes in some cases and conjunctive probability rating for both crimes in other cases, after all information had been presented. Overall, 360 probability ratings for guilt were collected from 120 participants, comprised of 40 judges, 40 attorneys and prosecutors, and 40 individuals without legal education. Our results provide evidence for a double conjunction fallacy (in this case, a higher probability of committing both crimes than the probability of committing either crime individually), in the group of individuals without legal education. These results are discussed in terms of their applied implications and in relation to a recent framework for understanding such results, quantum probability theory (QPT).

## Introduction

A fundamental principle in the judiciary system is that a judgment, decree, or decision rendered by a court and the interpretation, application, or enforcement of an existing law relating to a particular set of facts in some case are all products of rational, correct decision making. Such expectations for a rational standard in decision making increase with seniority in the judiciary system. For example, in the course of even a single case, a judge may have to make countless decisions, and every one of them is important for the overall proceedings and legal outcome. A judge decides if an accused stays out of jail pending trial, whether or not evidence is admissible, and what sources of information can be included in relation to a crime. Judges often decide if someone should be found guilty and sentenced, or may be placed on probation, and for how long. Judges must evaluate evidence, in accordance to particular (often very complex) rules, classify evidence, and employ evidence in order to issue a verdict. Attorneys and prosecutors must likewise have sufficiently high familiarity with legal proceedings, even if the corresponding expectations for normative decisions are not as high as for judges, but even lay individuals are sometimes expected to be able to operate at such high standards in legal proceedings (e.g., when members of a jury). There is a clear need to appreciate the psychological strengths and limitations of human minds to achieve the normative standard expected in relation to legal decision making (Gigerenzer and Goldstein, 1996). If human input into the judiciary process can undermine the accuracy of criminal verdicts, then the process may be ill equipped to distinguish truth and error and fall short of delivering the precision that befits the solemn epistemic demands of the criminal justice system and the certitude it proclaims to embody (Simon, 2012). Therefore, it is important to study and understand the cognitive processes involved in the evaluation of findings and evidence, probability assessment, problem solving, biases associated with legal decision-making, and how and when participants in legal proceedings rely on the perceptions of others (Kapardis, 2003; Daftary-Kapur et al., 2010; Spellman and Tenney, 2010).

Most of the previous research on legal decision making has focused on the influence of external factors, such as: pre-trial publicity, inadmissible evidence, scientific evidence, racial stereotypes, the confidence of eyewitnesses, or indeed their attractiveness and any informant's confidence in the quality of their knowledge (Spellman and Tenney, 2010; Fox et al., 2011; McCabe and Krauss, 2011; Tenney et al., 2011). Studies have shown that attorneys and prosecutors often have difficulty in judging whether a witness correctly identified the alleged perpetrator, memories are accurate, and a confession offers a truthful account of the suspect's deeds (Simon, 2012). Research in simulated and real legal settings has shown that an adequate model of judiciary decision making must include the role of human bias (Green and Wrightsman, 2003), for example, judges tend to explain a defendant's actions reflecting on their own past experiences and assumptions (Saks and Thompson, 2003). Likewise, there is evidence that attorneys and prosecutors underutilize probabilistic information and fail to understand a wide variety of statistical principles as well as methodological issues (McAuliff et al., 2003; Daftary-Kapur et al., 2010; Spellman and Tenney, 2010). Because of the influence of personal biases, attorneys and prosecutors given identical information may reach widely differing verdicts (Green and Wrightsman, 2003). For example, it has been found that final pieces of evidence have pervasive impact, the party going with argument as second is strongly advantaged and evidence occurring toward the end of the presentation is favored (Walker et al., 1972). In a series of studies Furnham (1986) has replicated recency effect in the formation of judgments of innocence and guilt of a defendant in an actual trial. Identical information received in a different order produced significantly different final verdicts and the effect generalized over population and time, that is different subjects yielded substantially different results. Such results are overall consistent with research across many more specific areas in decision making, showing that decisions are subject to framing or order effects and other apparently incidental aspects of the presented information and the evaluation process (e.g., Tversky and Kahneman, 1974; Hogarth and Einhorn, 1992).

Naturally, before a pattern of human decision making can be relegated to a non-normative bias, one needs a standard for normative decision making. Psychologists generally endorse classical probability theory (CPT; e.g., Kolmogorov, 1933) as the absolute standard for normative behavior (Oaksford and Chater, 2009; Tenenbaum et al., 2011), over and above notably classical logic (for the very interesting debate of what is the more appropriate foundation for rationality in human behavior see e.g., Oaksford and Chater, 2009, as well as Baratgin et al., 2014; Cruz et al., 2015; Politzer and Baratgin, 2016). The CPT axioms are a simple set of principles (e.g., see Howson and Urbach, 1993). These principles are identical whether probabilities are interpreted as frequencies or subjective degrees of belief. If one interprets probabilities as frequencies, then one effectively obtains a picture of probabilities using parts of generalized volumes, and one is led to the axioms of classical probability theory. If subjective degrees of belief are employed, these same axioms are derived with the aid of the Dutch book theorem (de Finetti et al., 1993). That is, the identical framework of CPT can be derived either from frequentist probabilities (via set theory) or subjective probabilities (with the aid of the Dutch book theorem). Psychologically, the normative case of CPT is supported by powerful mathematical results, notably the Dutch book theorem, according to which probabilistic assignment consistent with CPT protects from certain loss (de Finetti, 1993; Pothos et al., 2017). CPT is relevant in any situation where a person needs to adjust subjective probabilities, in light of existing and new evidence. Indeed, there have been several successful cognitive models based on CPT, providing good evidence that humans are mostly rational (overview in Oaksford and Chater, 2009). Equally, there have been persistent discrepancies between the prescription of CPT and human behavior and such results cast doubt for any strong claim regarding human rationality in decision making. Such results are often called fallacies, to emphasize that corresponding decision making is incorrect.

One of the most famous fallacies is the conjunction fallacy (CF), discovered by Tversky and Kahneman's (1983) in one of the most influential studies in decision making. Tversky and Kahneman presented brief vignettes to participants describing various hypothetical persons. In one of their examples, Linda was presented as intelligent and outspoken. Subsequent to reading the vignette, participants were asked to rank order several statements about Linda, in terms of how likely they considered them to be. The critical statements were that Linda is a feminist (F; very likely given the description), Linda is a bank teller (BT; very unlikely given the description), and Linda is a bank teller and feminist. Tversky and Kahneman reported results which indicated that *Prob*(*F*) > *Prob*(*BT&F*) > *Prob*(*BT*). The finding that *Prob*(*BT&F*) > *Prob*(*BT*) has been called the CF, as it is not possible in CPT. Before we consider how this comes about, note first that the CF has been extensively replicated—in both Tversky and Kahneman's original study and in subsequent work, there have been numerous replications: the CF is certainly not common in decision making, but at the same time it is easy to construct conditions that reliably lead to a CF (for a recent overview see Busemeyer et al., 2011).

There have been several attempts to reconcile the CF with CPT principles and rational behavior. An influential idea is to explain away the CF as a misunderstanding between what the experimenter intends and what the participant understands. Perhaps, in seeing the BT statement, participants understand *BT*&~F (which could be justified as a conversational implicature, Grice, 1975), another conjunction, and so one that can compare more flexibly with BT&F. There has been enormous research on this issue. The bottom line is that, even though various disambiguation procedures can reduce the rate of the CF, a residual CF is persistent (e.g., Dulany and Hilton, 1991; Sides et al., 2002; Moro, 2009; Tentori and Crupi, 2012). Different notions of probability may also impact on the extent to which a judgment should be considered a fallacy (Baratgin and Politzer, 2006).

Why is the CF so problematic for the assessment of rationality in human decision making? Consider the following problem. We are trying to estimate how often it rains and snows in December in Krakow. To do this, we consider all days on which it may have rained and/or snowed in Krakow in each December across the last 10 years. We count all the days on which it may have rained. From these rainy days, we identify the subset on which it snowed too. However, clearly it is not possible to have more days on which it both rained and snowed, than just rained. As noted, CPT implies a picture of probabilistic assignment as generalized volumes, with subsets/ events corresponding to parts of volumes—a conjunction is an intersection of conjuncts and it can never be greater than either conjunct.

There have been many attempts to absolve the CF of fallacy, even excluding any issues related to how the relevant statements are understood by participants. A trivial one is that when participants evaluate the conjunction they are in one frame of mind or context than when the evaluate the conjunct. Then, the CF result can be written by *Prob*(*BT* & *F*|*context*1) > *Prob*(*BT*|*context*2), and the conditionalizing variable no longer implies a fallacy. However, most decision researchers are not content with such explanations (we defer a more complete discussion of possible accounts of the CF until we have actually demonstrated a CF). Instead, a popular approach is to assume that there are two separate routes to decision making, one which is fully normative and produces decisions consistent with the principles of CPT and one which is quick and approximate, for quick relatively unimportant decisions, which is the norm in every day decision making (Sloman, 1996; Kahneman, 2001; DeNeys and Goel, 2011; Elqayam and Evans, 2013). Given the importance of decision making in legal contexts for our lives, such an approach would have to predict that, if humans are capable of normative decision making at all, this would be evidenced in such contexts—but, it also appears that decision fallacies can sometimes appear in such contexts (see also Saks and Thompson, 2003).

With the above considerations in mind, we consider whether the CF can be evidenced in decision making relating to legal problems, using participants across a wide range of legal expertise, from (legally) naïve individuals, to attorneys and prosecutors, to judges (in Poland); the latter category represents an expectation of the highest possible standard of legal decision making and, if there is reality to dual-route models of human decision making, they should embody the normative route more so than other groups. The experimental investigation is based on the presentation of information regarding two possible crimes different individuals may have committed. The information for each crime is presented sequentially. For a particular case, some participants had to decide whether suspects committed each crime individually, other participants had to make a single conjunctive judgment of whether the suspect was guilty of both crimes. All participants were presented with multiple cases, so that they had to provide probability ratings both for conjunctions and individual conjuncts. Classically, if one considers all these suspects, say they are 100, some of them would have committed only the first crime and some of them the second crime. For a suspect to have committed both crimes, clearly he/she needs to have also committed either crime—it is not possible for the probability of the conjunction (committing both crimes) to be greater than for either conjunct (committing either crime individually). However, psychologically, the logic of the design is that if a person is, for example judged to be guilty of one crime, then he/she would be likely to have committed the other too, especially in cases of related crimes (which is indeed close to one of Tversky and Kahneman's, 1983, assumptions for when to expect a CF, i.e., when there is some causal relation between the conjuncts).

## Materials and methods

### Participants

The study was approved by the ethical committee of the Institute of Psychology at the University of Silesia and all participants provided written consent prior to participation.

We briefly describe the legal system in Poland, since all participants were recruited in that country. According to the rules governing Polish criminal proceedings, the judge plays an active role in the trial when the evidence is examined. All courts are presided over by judges who are appointed by the President of the Republic on the recommendation of the National Council of Judiciary. The judges in Poland are independent and are accountable only to the law. The minimum age for appointment is 26 and the compulsory retirement age is 70. Candidates for judicial appointment must successfully pass a public exam and be employed for at least 2 years as an assistant judge. Professional judges must attain a university law degree and complete a 4-year training period and a 2-year period of court apprenticeship. Professional judges and lay assessors together deliberate and vote on the penalty to be imposed.

The public prosecutor is the only person who has the right to issue an order on the presentation of charges, to decide on the application of pre-trial detention, or to decide the method of terminating the proceedings. Defendants are presumed innocent until proven guilty; the burden of proof is on the prosecution. The accused have the right to a legal advisor, to argumentation, to participate in procedural actions, to appeal against procedural decisions, and to be acquainted with the case files. Accused persons have the right to employ a legal adviser (attorney or solicitor) for their defense, if the accused cannot afford to pay for one, they may be granted legal aid at public expense.

Court proceedings are usually public and take the form of an oral hearing that is documented on the record. Sentencing is decided immediately after the judges and lay assessors deliberate and vote on the question of guilt, the legal classification of the act, and other issues (e.g., civil complaints). This all occurs in one session. There is a separate evidentiary proceeding devoted to the question of the penalty, and a separate session with deliberation and voting on the penalty imposed. The judgement is an integral whole and is pronounced on the question of guilt and penalty at the same time. After the judgement is passed, it is immediately recorded in writing. In addition, the presiding judge must always announce it orally in the courtroom.

The law requires the judge and other members of the panel to rely on three factors during sentencing: (1) evidence and its evaluation, (2) the principles of science, and (3) personal experience. The confession of the accused is considered an important part of the evidence, but it is not sufficient in itself to prove guilt. The defendant may not be interrogated under oath. Unlike the Anglo-Saxon process, there are no separate presentations of evidence by the prosecutor and the defense during the trial. Expert opinion is subject to evaluation by the court according to the rules of evidence of Polish criminal procedure. For this reason, the court is not bound by the expert's conclusion. However, as in most other jurisdictions, expert opinions, especially given by psychiatrists, are accepted by the courts. This is particularly true if the offender's mental capabilities or potential dangerousness are assessed.

Three groups of participants took part in the experiment: 40 criminal court judges (22 women, 18 men, aged between 29 and 66 years, *M* = 42.25; *SD* = 7.66; with professional experience from 3 to 30 years, *M* = 13.5; *SD* = 6.30); 40 prosecutors and attorneys (20 women, 20 men; aged between 28 and 60 years, *M* = 40.45; *SD* = 8.35; with professional experience raging from one to 35 years, *M* = 10.05; *SD* = 8.18); and 40 participants without legal background (21 women, 19 men, aged between 21 and 62 years *M* = 32.30; *SD* = 11.09; their non-legal professional experience ranged from none to 40 years; *M* = 9.63; *SD* = 10.80). All participants were recruited in Poland and were Polish. The first author used his professional experience and network to identify professional participants (judges, prosecutors, and attorneys) willing to take part. The participants with no legal background were recruited by the first author as well, amongst colleagues/ acquaintances. Decision as to whether or not to take part in this study was completely voluntary. Subjects were informed, that the aim of the research is to explore legal decision making and distinctive characteristics of judges and lawyers in evidence evaluation mechanisms. Participants received a consent form (for participation to the study) prior to the study and a debriefing form after the end of the study. Participants received no compensation or remuneration for taking part in the research. Note, given the focus of the study on judges and attorneys in law (including prosecutors), the only option for proceeding was professional/personal contacts of the first author (who is a practicing attorney). Notably, we could not offer financial incentives for participation, because it is extremely complicated in the Polish system to pay judges or prosecutors (and indeed it is unlikely that a small payment would motivate such individuals to take part in a psychology study). Given that one part of the sample was not monetarily compensated for participation, it was appropriate to seek volunteers for all participant groups. One might wonder whether the voluntary nature of participation could introduce systematic biases in participants' performance, but in this case it is unclear what would be the nature of such biases (this is not a study where higher motivation could easily translate to a simple measure of “better” or worse performance, at least not in a way that would be obvious to participants; Orne, 1962). Still, we cannot preclude the possibility that the personal connection that was the basis for recruitment might have influenced results in a certain way.

### Design

**Figure 1:**
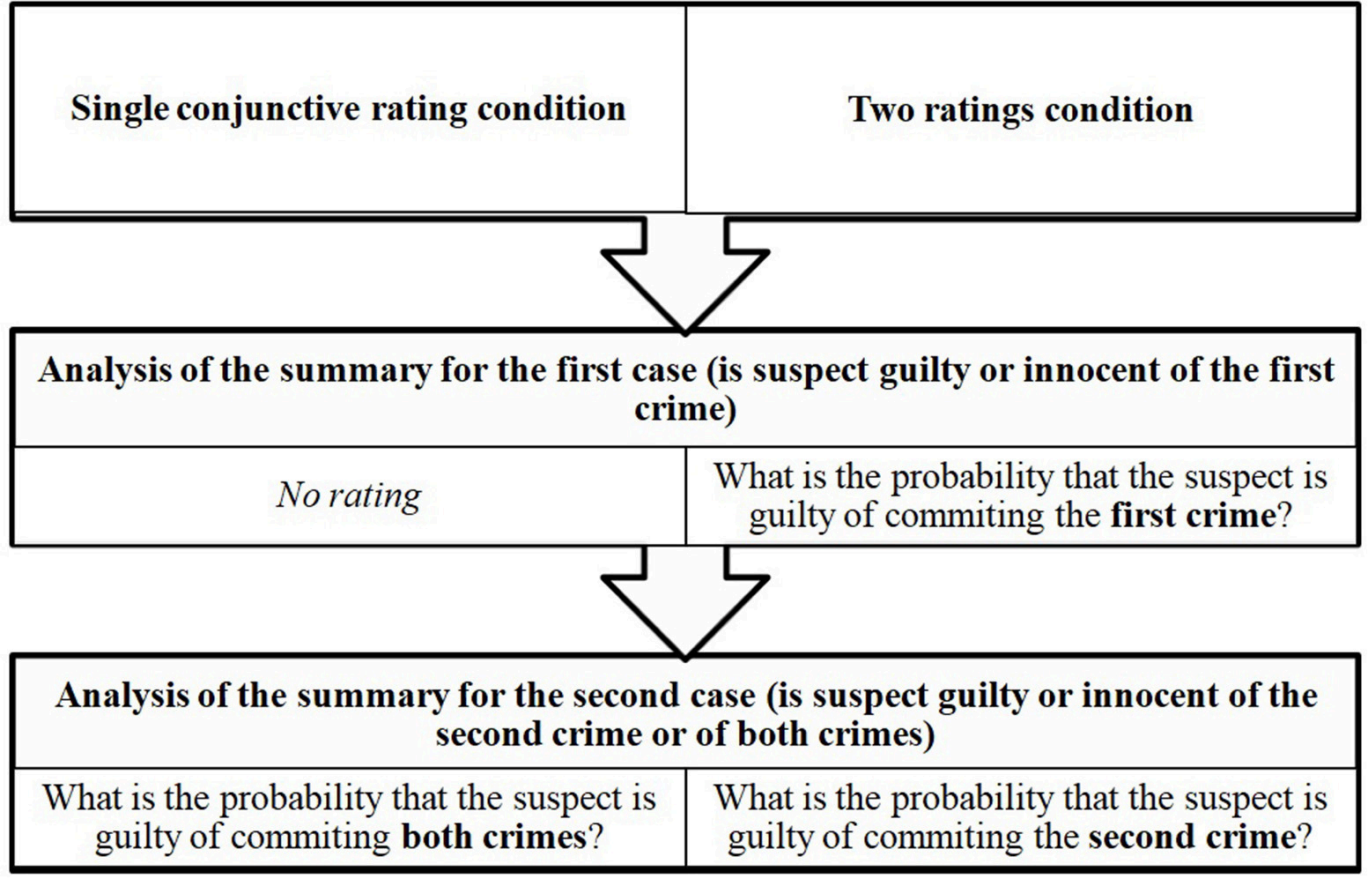
The design of the study and an outline of the procedure.

The experiment had a 2 (is the suspect guilty or innocent?: guilty vs. innocent) x 2 (rating: a single conjunctive rating for both crimes vs. two ratings for each crime) x 3 (participant: judge vs. lawyer vs. layperson) mixed design (Figure 1). The participant variable was a between participants factor, the suspect and rating variables were within participant factors. The CF would be committed if there is a main effect of rating. Note, for a particular case, a CF would be assessed on the basis of ratings from different participants, but each participant provided ratings for both individual crimes and conjunctive ratings for the two crimes in a case. The two challenges we had to address are these: First, we wanted participants to make judgments both for conjunctive and individual events. This is an obvious requirement since the probabilistic assessment for e.g., a conjunction matters only by comparison to that of a marginal. Second, it is clearly meaningless to ask the same participant to rate simultaneously both the conjunction and the individual statements for the same case. Equally, we could not adopt Tversky and Kahneman (1983) ingenious procedure of rank ordering marginals and conjunctions. Therefore, we resorted to a mixed effects approach, with specific case tracked explicitly in the analyses (instead of averaging). Regarding the conceptual assumptions that support a conclusion of a CF or not, for each case we took care to provide detailed vignettes with all relevant information so that, *to a first approximation*, all participants can be thought of as having the same knowledge prior to providing probability estimates.

An interesting possibility concerns an interaction between rating and participant, with some participant groups less likely to commit the CF. Dual route approaches to decision making will lead us to expect that judges would be less likely to commit the CF, compared to participants with no legal training.

### Materials

During previous studies we have gained access to real life criminal cases records from different District Courts and Appellate Courts in Poland of criminal investigations concerning crimes committed between years 2000 and 2015. The files for each criminal case, including interrogation and interview protocols, expert evidence, and adjudications, were thoroughly reviewed and photocopied. Out of these 400 criminal cases, we first selected 60 cases based on the following criteria. First, we chose records from different court districts than those of the (legal profession) participants to eliminate possibility that subjects will be assessing cases they would have come across in the past. Second, we aimed for an approximate balance between cases in which the suspect was guilty and innocent, specifically cases for which the suspect was innocent of both crimes and guilty of both. Guilt of the suspect in individual cases was established on the basis of the ruling court's justification. Third, we required that, in cases of true statements from the suspect, there was overwhelming evidence independent of the suspect's statement which corroborated the statement and, in cases of false statements, that there was overwhelming evidence independent of the suspect's statement which refuted it. These criteria for inclusion are reasonable given that, on the one hand, complete truths are usually unobtainable in criminal cases but, on the other hand, making correct classifications of the transcripts was crucial for the study's validity. Fourth, as the study was based on case summaries (it would be impractical to employ full records), we ensured that the case summaries correctly revealed the guilt or innocence of the suspect. This was established by having two independent competent raters—an experienced retired prosecutor and an experienced retired attorney recruited by the experimenter—examine each summary and determine whether the summary led to a conclusion regarding the suspect's guilt and innocence for one or both offenses consistent with the trial outcome. Only case summaries of confirmed valence during this preliminary stage were used in the main experiment. Finally, factors such as the type of crime and the suspect's gender, age, and ethnicity varied across the transcripts (but could not be fully counterbalanced). The above criteria allowed us to identify 18 case summaries (from the original 60 cases), divided as follows: 9 summaries of cases when suspect was guilty of both charges and 9 summaries of cases when suspect was innocent of both charges. All the materials were in Polish and are available from the authors on request.

### Procedure

All participants received three criminal case summaries to assess. They could freely choose where and when to complete the task. Participants were instructed to read the descriptions carefully and to assign a probability that the suspect is guilty on a 10-point scale, with anchors: 1-definitely innocent, to 10-definitely guilty. Note, arguably in real life legal decision making judgments are qualitative, but an examination of putative CFs requires numerical estimates (but see Politzer and Baratgin, 2016, for a broadly relevant alternative approach). It took the subjects from 20 to 50 min to read descriptions of individual cases and to assign the probability. For each case, participants either rated the probability of each crime separately (two single ratings for each crime) or they provided a single conjunctive rating for both crimes. Participants randomly received two vs. one cases in which they had to make two individual judgments vs. one conjunctive one, or vice versa. So, each participant would have to make between four and five guilt determinations. Presentation order was randomized across participants, so that for the same pair of crimes, some participants were shown the relevant information in one order and other participants in the converse order.

## Results

Unless stated otherwise, the dependent variable was the rating that a suspect was guilty of a particular crime, denoted as *Prob*(guilt). We collected 360 ratings from participants, equally divided between the three groups of participants (judges, lawyers, individuals without a legal background). Of these ratings, 170 pairs of crimes were rated individually and 190 pairs of crimes were rated with a single conjunctive rating. Subjects assigned probability of suspect's guilty on a 10-point scale, with anchors: 1-definitely innocent, to 10-definitely guilty. Below, when we talk about probability of guilt, we imply a linear mapping from these ratings to assumed, subjective probabilities.

We ran a mixed effects model (multilevel linear model in SPSS with both random and fixed effects) with probability rating as the dependent variable, profession as a fixed effect between participants independent variable (three levels: judge, lawyer, participants without legal education), rating type as a fixed effect within participants independent variable (three levels, probability for A, probability for B, and probability for the conjunction A&B), and case type (two levels, innocent vs. guilty cases) as the last fixed effect independent variable. Two interaction terms were included, rating x profession and rating x profession x case type; the restriction to these interaction terms was theoretically motivated. Note, it may be thought that case type should actually be used to split the data file, since obviously ratings for innocent cases would be very different for ratings for the guilty ones. However, regarding the key hypothesis of whether a CF occurs or not, for both guilty and innocent cases a CF involves probabilities in the same direction, *Prob*(*A&B*) > *Prob*(*A*) or *Prob*(*A&B*) > *Prob*(*B*) (or both). We employed a single random effect of participant. The random effect was modeled with both intercept and slopes, but random slopes were employed only for the case type fixed effect, partly so as to not overcomplicate the model, partly because theoretically it is mainly the relationship between case type and the dependent variable that might plausibly be affected by the participant random effect. Note, the possibility of introducing a second random effect of particular test item is problematic because crossing these two random effects (participant and specific item) leads to many cells with just one observation.

An unstructured covariance matrix was assumed and model parameters were estimated with maximum likelihood, so that −2 log likelihood could be employed to evaluate nested models. For brevity we only consider three models, one without the random effect, one with the random effect but only intercepts, and the final one with the random effect and both intercepts and slopes. Each model elaboration was highly significant so it was the final model that was employed in the assessment of the hypotheses of interest [χ^2^(1) = 10, *p* = 0.001; χ^2^(5) = 55, *p* < 0.0005]. For the final model, there was a highly significant main effect of case type [*F*_(1, 171.5)_ = 403, *p* < 0.0005], which is hardly surprising, since all it indicates is that participants responded differently to the cases which were really for guilty suspects vs. ones which were really for innocent suspects. This main effect is simply a validation of the design. The mean rating for guilty cases was indeed higher than for innocent ones [M_Guilty_ = 8.935, SE_Guilty_ = 0.121 vs. M_Innocent_ = 3.995, SE_Innocent_ = 0.204). More interestingly, there was also a highly significant triple interaction between rating, profession, and case type [*F*_(8, 207.2)_ = 2.706, *p* = 0.007].

**Figure 2:**
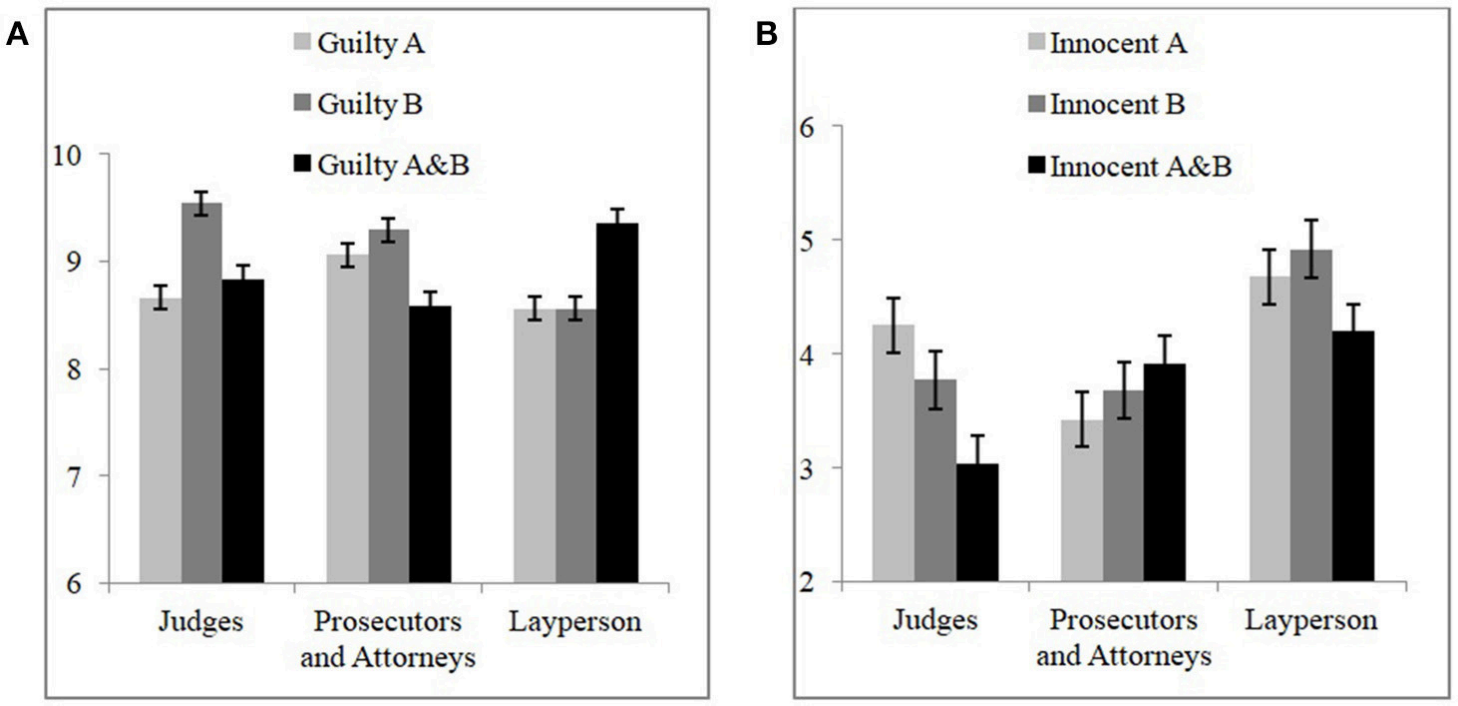
CF and guilt probability ratings by different professional groups of **(A)** guilty suspects' cases and **(B)** innocent suspects' cases (on a 1 to 10 points scale), bars represent standard error of the mean.

It is by analyzing this triple interaction that we can assess evidence for a CF, for different professions, and separately for the guilty vs. innocent cases. In order to do this, we ran simpler mixed effects models with participant still included as a random effect, but now the influence of the random effect restricted to intercepts; there was only a single fixed effect of rating. The *post-hoc* procedure for estimated means was uncorrected *t*-tests, since in all cases such comparisons would be planned as tests of a putative CF. For when the case type was guilty, for judges there was an effect of rating [*F*_(2, 64.3)_ = 3.503, *p* = 0.036], but no evidence for a CF, rather a situation where *Prob*(*B*) > *Prob*(*A&B*) (M_ProbB_ = 9.543, SE_ProbB_ = 0.250; M_ProbA&B_ = 8.831, SE_ProbA&B_ = 0.276, marginally approaching significance, *p* = 0.058, d = 0.612) (Taylor, 2015). For lawyers there was no main effect of rating [*F*_(2, 94)_ = 2.331, *p* = 0.103]. For participants without a legal background there was an effect of rating [*F*_(2, 61.9)_, *p* = 5.273, *p* = 0.008] and, importantly, in this case there was evidence for a double CF with *Prob*(*A&B*) (M_ProbA&B_ = 9.354, SE_ProbA&B_ = 0.228) being greater than both *Prob*(*A*) (M_ProbA_ = 8.563 SE_ProbA_ = 0.217) and *Prob*(*B*) (M_ProbB_ = 8.563, SE_ProbB_ = 0.217; in both cases *p* = 0.005, d = 0.636). For when the suspect was really innocent, there was no effect of rating for judges [*F*_(2, 58.6)_ = 2.081, *p* = 0.134] or for lawyers [*F*_(2, 72)_ = 0.306, *p* = 0.737], and in this case no effect for participants without a legal education too [*F*_(2, 58.4)_ = 1, *p* = 0.375]. Figure 2 presents the probability assessments of judges, lawyers, and participants without legal background.

## Discussion

A CF was observed in the case of probabilistic assessment in legal decision making, but only for participants without a legal background. Participants rated the probability of guilt for a suspect accused of two crimes. Participants either rated each crime separately or provided a single conjunctive judgment for both crimes. The finding that a CF can be observed in legal decision making is perhaps less surprising, since there have been other instances of probabilistic errors in related contexts (Walker et al., 1972; Furnham, 1986). The important conclusion concerns the lack of a CF, for participants with a legal background.

The observation of a double CF in the participant group with no legal background, but not for attorneys in law or judges, raises a potential question of whether the presence of the double CF in legal decision making of the type we explored has to do with lack of a legal background specifically or lack of a professional background more generally. The evidence suggests it is the former, since we took care to match the non-legal profession participants as closely as possible with the participants having a legal background (e.g., the average professional experience of the non-legal background participants was 9.84 years). Of course, one can wonder whether analogous patterns would be observed in other, specific professional groups, e.g., doctors. However, such an objective is beyond the scope of this work (and perhaps of arguable interest given the already extensive evidence for CF; e.g., see Busemeyer et al., 2011, for a review). Instead, our question was this: considering a legal decision making situation where professional participants without a legal background can demonstrate a (double) CF, is there evidence that participants in legal professions demonstrate a similar bias? The answer is no and we argue that this is an interesting conclusion exactly because of the higher expectations for normative (legal) decision making for agents in theses professions.

Now that we have observed a CF, we turn the discussion to formal accounts of the CF. Some of the related formal work has been directed toward assessing whether a CF indeed represents a probabilistic error. Recently, there have been a few attempts to recast the CF as a judgment consistent with probability theory. We highlight two such attempts and focus on a third. First, inductive confirmation proponents have argued that in CF experimental paradigms participants do not mentally compute a conjunction, when faces with a statement such as BT&F (in Tversky and Kahneman's, 1983, Linda paradigm), but rather with inductive confirmation, the extent to which adding some information (e.g., the Linda story) corroborates or disconfirms a hypothesis (Tentori et al., 2013). Inductive confirmation is certainly a very powerful idea in belief updating, however, there are some concerns as to whether a CF paradigm can be recast as an inductive confirmation problem, especially in the case of the present experiment, where the conjunctive nature of the decision on both crimes was clear (see also Busemeyer et al., 2015b). Second, some researchers have advocated a view of normative decision making based on CPT, but with noise (Costello and Watts, 2014). According to this view, probabilistic inference involves a process of mental sampling/ simulation, which is more error prone for more complex probabilities, such as conjunctions. However, there is no directly empirical evidence for such a mental sampling process and indeed the assumption appears particularly suspect in the case of highly unique and individualized situations, such as the crime summaries in the present study. Additionally, there have been some concerns regarding the extent to which the noise approach can offer a coherent account of both conditional probabilities and order effects in decision making.

Quantum probability theory (QPT) is a third formal approach to the CF and, even though it is not without criticism (Busemeyer and Wang, 2015; Boyer-Kassem et al., 2016), it does have an advantage of generality and generative value (e.g., Pothos and Busemeyer, 2013; Wang et al., 2014; White et al., 2014; Bruza et al., 2015). We explain the QPT account detail since, while it can provide a simple and intuitive explanation for single CFs, double CFs require a degree of elaboration. We call QPT the rules for how to assign probabilities to events from quantum mechanics, without any of the physics. QPT basically provides a set of rules for probabilistic inference, alternative to CPT. QPT cognitive models have been pursued exactly for empirical results which are problematic from a classical perspective, such as the CF. They have been employed to accommodate a fairly wide range of decision fallacies, including order effects in evidence assessment in legal decision making (Aerts and Aerts, 1995; Pothos and Busemeyer, 2009; Trueblood and Busemeyer, 2010, 2011; Busemeyer et al., 2011; Wang et al., 2014; White et al., 2014, 2016; overviews in Busemeyer and Bruza, 2011; Haven and Khrennikov, 2013; Pothos and Busemeyer, 2013). QPT inference is strongly dependent on context and perspective, so that, for example, the nominally same question may have different meanings depending on whether it is asked in isolation or together with other questions. Another feature of QPT is that some questions are compatible and for such questions probabilistic picture is nearly the same as in the classical case. However, other questions are incompatible and incompatible questions are subject to strong contextual/ order effects.

**Figure 3:**
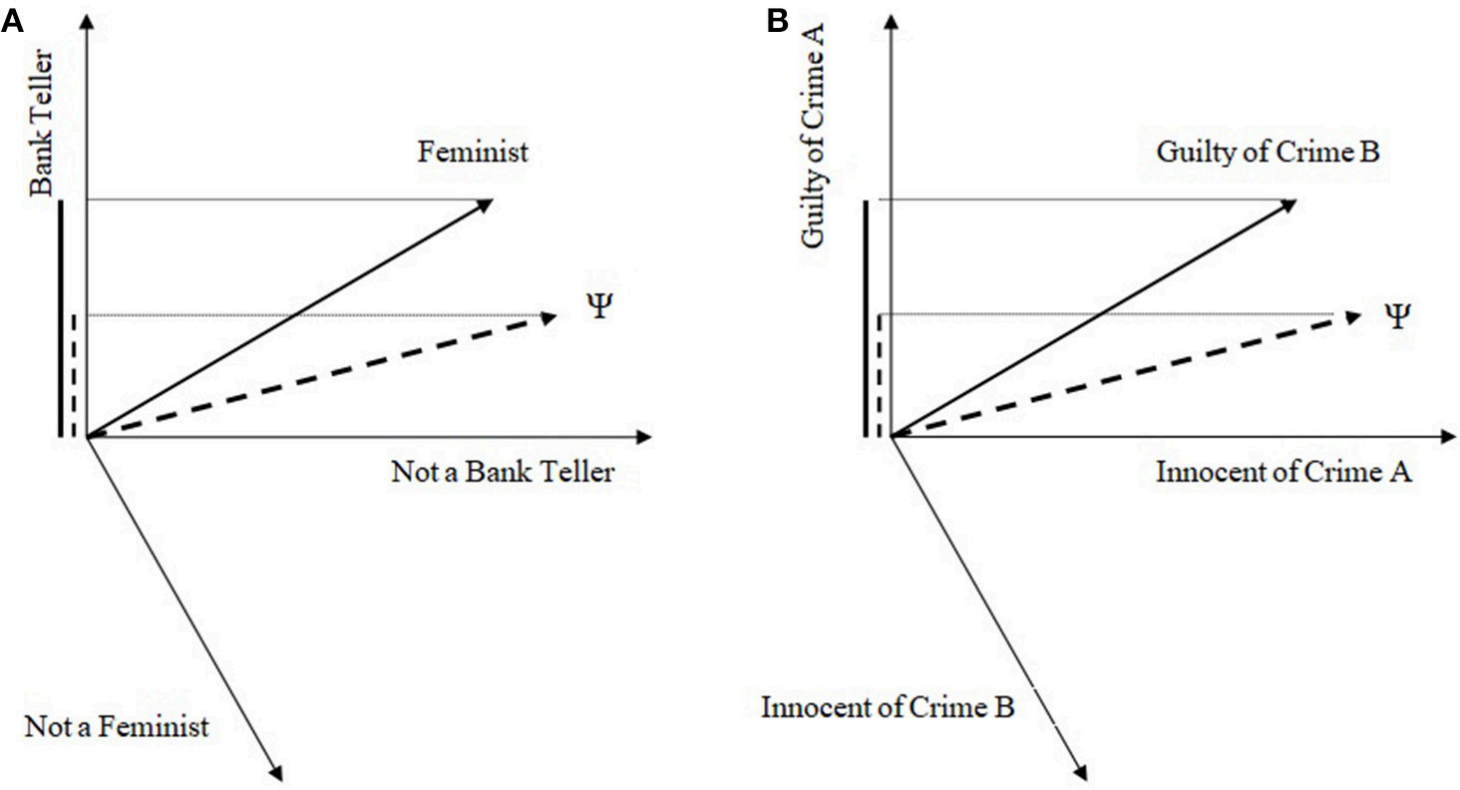
An illustration of the QP approach for the (single) conjunction fallacy in **(A)** the classic “Linda problem” and in **(B)** legal decision making.

In the case of a single CF, we can illustrate a QPT approach by analogy to Busemeyer et al.'s (2011) quantum model for the standard CF. The illustration develops on the basis of a greatly simplified caricature of the full model, but this is sufficient for the present purposes. In QPT, the first step is to specify a vector space such that each subspace in this space corresponds to the outcomes of different questions. In the simplest case, these subspaces are one dimensional (rays). The second step is to determine a (normalized) vector which has the role of the mental state just prior to answering the questions. Note, in QPT probabilistic assessment is always (mental) state dependent, while for CPT the role of the mental state, if at all, has to be taken into account as conditionalizing information. The third step is to compute probabilities given the QPT rules: the probability that a participant in mental state represented by ψ answers with “yes” a binary question A is given by $Prob\left(A;\psi \right)={\left|P_{A,yes}\psi \right|}^{2}$, where *P*_*A, yes*_ is the projector operator onto the subspace representing the yes outcome for question A. A projector operator takes a vector and lays it down onto a subspace; then, probability is computed as the squared length of this projection. In the case of the classic CF, an illustrative caricature of the model is simply as in Figure 3A, which was constructed by taking into account the minimal assumptions that the mental state at the outset has to be closer to the F_yes_ ray than the BT_yes_ one and that the BT, F rays are relatively uninformative relative to each other (a prototypical F is not particularly likely or unlikely to be a BT and vice versa). Then, $Prob\left(BT;\psi \right)={\left|P_{BT,yes}\psi \right|}^{2}$, $Prob\left(F\&\;then\;BT;\psi \right)={\left|P_{BT,yes}P_{F,yes}\psi \right|}^{2}$. Note first that a conjunction for QPT incompatible questions can only be computed as a sequential projection, since we cannot concurrently determine the truth/falsity of incompatible questions. It can be readily seen that the end result from the sequential projection *P*_*BT, yes*_*P*_*F, yes*_ψ is larger than *P*_*BT, yes*_ψ, that is, *Prob*(*F& then BT*; ψ) > *Prob*(*BT*; ψ). Without going into the details of whether this is a good model for the CF in the Linda paradigm or not (see Busemeyer et al., 2011, 2015b), one can readily see that it is possible to obtain the CF as a “correct” probabilistic outcome. The conceptual interpretation of the QPT CF model is that from the initial perspective of the Linda story it is very difficult to see Linda as a BT, but once we accept Linda as a F, then it becomes easier to imagine her as a BT too.

The reason for considering the above QPT approach is that this is the one most commonly employed in considerations of how QPT can describe the CF (Busemeyer et al., 2015a,b). However, most existing research in the CF has focused on single CFs, according to which *Prob*(A) > *Prob*(A&B) > *Prob*(B), that is a CF occurs only for one of the two conjuncts. If we had observed single CFs in the present results as well, it would be straightforward to adapt existing QPT work (e.g., Figure 3B). Instead, the main result we obtained was one of a double CF. It is important to explain why the above approach cannot accommodate a double CF. For a double CF, we have: *Prob*(A), *Prob*(B) < *Prob*(A&B), that is a CF occurs for both conjuncts. That the above QPT approach cannot accommodate double CFs can be seen by noting that $Prob\left(F\&\;then\;BT;\psi \right)={\left|P_{BT,yes}P_{F,yes}\psi \right|}^{2}={\left|P_{BT,yes}\psi_{F,yes}\right|}^{2}{\left|P_{F,yes}\psi \right|}^{2}=Prob\left(BT|F\right)Prob\left(F\right)$. Therefore while we can have *Prob*(*F& then BT*; ψ) > *Prob*(*BT*; ψ), it is not possible to also have *Prob*(*F &then BT*; ψ) > *Prob*(*F*; ψ).

The double CF in the present work in the present work is an important empirical finding partly for the above reasons. It is worth noting that double CF evidence in the literature generally has been controversial (e.g., Busemeyer et al., 2015b), though in the present case a double CF is intuitive. For the participants with no legal background, we have a situation where *Prob*(A), *Prob*(B) < *Prob*(A&B), for when evaluating criminal cases for which the suspect was guilty for both crimes.

Given that the standard version of an influential approach for the CF, QPT, cannot accommodate a double CF, it is worth exploring how a QPT account could be extended. A starting point is that the representation for such cases involves compatible questions, rather than incompatible ones as above—this is an important assumption and we will return to it. This means that the consideration of each crime takes place in a separate QPT space. If in addition we have a tensor product representations along the lines ψ = |_*c*1_*yes*_*Crime*1*yes*_ + *c*1_*no*_*Crime*1_*no*_ 〉 ⊗ |_*c*2_*yes*_*Crime*2*yes*_ + *c*2_*no*_*Crime*2_*no*_ 〉 = *c*1_*yes*_*c*2_*yes*_ |*Crime*1_*yes*_ 〉 |*Crime*2_*yes*_ 〉 + *c*1_*yes*_*c*2_*no*_ |*Crime*1_*yes*_ 〉 |*Crime*2_*no*_ 〉 + *c*1_*no*_*c*2_*yes*_ |*Crime*1_*no*_ 〉 |*Crime*2_*yes*_ 〉 + *c*1_*no*_*c*2_*no*_ |*Crime*1_*no*_ 〉 |*Crime*2_*no*_ 〉 (where |… 〉 is the Dirac bracket notation, indicating column vectors; we omit the tensor product symbol ⊗ after the first expression for brevity, and *c*1_*yes*_, *c*1_*no*_, *c*2_*yes*_, *c*2_*no*_ are complex coefficients), then the situation is mostly classical. Denote as *P*_*Crime*1*yes*_ the projector for a “yes” response for the first crime in a case and analogously for other P … objects. For example, $Prob(Crime1_{yes}\&Crime2_{yes};\psi )=|P_{Crime1yes}⊗P_{Crime2yes}\psi {|}^{2}=|P_{Crime1yes}⊗P_{Crime2yes}c1_{yes}c2_{yes}\;|Crime1_{yes}\rangle \;|Crime2_{yes}\rangle {|}^{2}=|c1_{yes}c2_{yes}{|}^{2}=|c1_{yes}{|}^{2}|c2_{yes}{|}^{2}=Prob(Crime1yes)Prob(Crime2yes)$, as expected classically. However, not all states ψ can have a tensor product structure. An extreme case of so-called entanglement would be a state of the form ψ = *a* |*Crime*1_*yes*_ 〉 |*Crime*2_*yes*_ 〉 + *b* |*Crime*1_*no*_ 〉 |*Crime*2_*no*_ 〉, implying a commitment to a situation that a suspect either definitely committed both crimes or definitely did not commit either crime. In such a state, deciding on the first crime resolves any uncertainty about the second crime too. However, this is not sufficient to produce a CF. If, for example, we write ψ = *a* |*Crime*1_*yes*_ 〉 |*Crime*2_*yes*_ 〉 + *b* |*Crime*1_*yes*_ 〉 |*Crime*2_*no*_ 〉 +*c* |*Crime*1_*no*_ 〉 |*Crime*2_*yes*_ 〉 +*d* |*Crime*1_*no*_ 〉 |*Crime*2_*no*_ 〉, then $Prob\left({Crime1}_{yes};\psi \right)={\left|P_{Crime1yes}⊗I\psi \right|}^{2}={\left|a\right|}^{2}+{\left|b\right|}^{2}$ and $Prob\left({Crime1}_{yes}\;\&\;{Crime2}_{yes};\psi \right)={\left|P_{Crime1yes}⊗P_{Crime2yes}\psi \right|}^{2}={\left|a\right|}^{2}$. Clearly, *Prob*(*Crime*1_*yes*_; ψ) > *Prob*(*Crime*1_*yes*_ &*Crime*2_*yes*_; ψ).

The observed results motivate the consideration of an initial representation for the mental space in a tensor product structure as above, but also a thought process which “mixes” thoughts and beliefs between the two crimes (Pothos and Busemeyer, 2009; Broekaert et al., 2017). It is not our purpose presently to outline in detail a full cognitive model for the consideration of criminal cases and we focus on the technical elements of QPT potentially relevant for coverage of the results (for more relevant details see Pothos and Busemeyer, 2009; Trueblood and Busemeyer, 2011; Pothos et al., 2013; Wang et al., 2013; Narens, 2014). The generic thought process in QPT is typically modeled as a unitary operation and the specification of the unitary embodies the psychological assumptions about the thought process (however, note that analogous constructions in CPT still have to conform to the conjunction constraint; Pothos and Busemeyer, 2009, demonstrate this directly). The unitary can have a tensor product structure *U*_*Crime*1_ ⊗ *U*_*Crime*2_, in which case nothing changes relative to above, $Prob\left({Crime1}_{yes}\&{Crime2}_{yes};\psi \right)={\left|P_{Crime1yes}⊗P_{Crime2yes}U_{Crime1}⊗U_{Crime2}\psi \right|}^{2}={\left|P_{Crime1yes}U_{Crime1}⊗P_{Crime2yes}U_{Crime2}\psi \right|}^{2}$; this final expression will be constrained by the conjunction constraint. Suppose though that U has a more complex form, for example, $U=\left(\begin{array}{cc} \begin{array}{cc} 1 & 0\\ 0 & 0 \end{array} & \begin{array}{cc} 0 & 0\\ -1 & 0 \end{array}\\ \begin{array}{cc} 0 & -1\\ 0 & 0 \end{array} & \begin{array}{cc} 0 & 0\\ 0 & 1 \end{array} \end{array}\right)\neq U_{1}⊗U_{2}$ for any individual *U*_1_, *U*_2_. The main characteristic of this unitary is that it “mixes” amplitude from the space corresponding to the consideration of one crime to the space of the other. It is convenient to revert to matrix notation here, writing $\psi =\left(\begin{array}{c} \begin{array}{c} a\\ b \end{array}\\ \begin{array}{c} c\\ d \end{array} \end{array}\right),P_{Crime1yes}⊗I=\left(\begin{array}{cc} 1 & 0\\ 0 & 0 \end{array}\right)⊗\left(\begin{array}{cc} 1 & 0\\ 0 & 1 \end{array}\right)=\left(\begin{array}{cc} \begin{array}{cc} 1 & 0\\ 0 & 1 \end{array} & \begin{array}{cc} 0 & 0\\ 0 & 0 \end{array}\\ \begin{array}{cc} 0 & 0\\ 0 & 0 \end{array} & \begin{array}{cc} 0 & 0\\ 0 & 0 \end{array} \end{array}\right)$, $I⊗P_{Crime2yes}=\left(\begin{array}{cc} 1 & 0\\ 0 & 1 \end{array}\right)⊗\left(\begin{array}{cc} 1 & 0\\ 0 & 0 \end{array}\right)=\left(\begin{array}{cc} \begin{array}{cc} 1 & 0\\ 0 & 0 \end{array} & \begin{array}{cc} 0 & 0\\ 0 & 0 \end{array}\\ \begin{array}{cc} 0 & 0\\ 0 & 0 \end{array} & \begin{array}{cc} 1 & 0\\ 0 & 0 \end{array} \end{array}\right)$, and $P_{Crime1yes}⊗P_{Crime2yes}=\left(\begin{array}{cc} 1 & 0\\ 0 & 0 \end{array}\right)⊗\left(\begin{array}{cc} 1 & 0\\ 0 & 0 \end{array}\right)=\left(\begin{array}{cc} \begin{array}{cc} 1 & 0\\ 0 & 0 \end{array} & \begin{array}{cc} 0 & 0\\ 0 & 0 \end{array}\\ \begin{array}{cc} 0 & 0\\ 0 & 0 \end{array} & \begin{array}{cc} 0 & 0\\ 0 & 0 \end{array} \end{array}\right)$.

Given the above specification, we have:

$$\begin{array}{l} Prob\left(\text{​}Crime1_{yes};\psi \text{​}\right)=\text{​}P_{Crime1yes}⊗\;I\cdot U\cdot \psi^{2}\\ \;=\text{ ​}|\text{​}\left(\begin{array}{llll} 1 & 0 & 0 & 0\\ 0 & 1 & 0 & 0\\ 0 & 0 & 0 & 0\\ 0 & 0 & 0 & 0 \end{array}\right)\cdot \text{ ​}\left(\begin{array}{llll} 1 & 0 & 0 & 0\\ 0 & 0 & -1 & 0\\ 0 & -1 & 0 & 0\\ 0 & 0 & 0 & 1 \end{array}\right)\text{ ​}\cdot \text{ ​}\left(\begin{array}{l} a\\ b\\ c\\ d \end{array}\right)\text{ ​}{|}^{2}\text{​}=\text{​}|a{|}^{2}\text{​}+\text{​}|c{|}^{2} \end{array}$$

$$\begin{array}{l} Prob\left(Crime2_{yes};\psi \right)=|I⊗\;P_{Crime2yes}\cdot U\cdot \psi {|}^{2}=|a{|}^{2}+|b{|}^{2}\\ \;Prob(Crime1_{yes}\&Crime2_{yes};\psi )=|a{|}^{2} \end{array}$$

Recall that a mental state vector in QPT is normalized, therefore |*a*|^2^ + |*b*|^2^ + |*c*|^2^ + |*d*|^2^ = 1. But it should be clear that this scheme still cannot accommodate a CF, which illustrates that only certain space structures can produce a single CF (e.g., as in Pothos and Busemeyer, 2009) and it is unclear whether a double CF is possible at all.

Overall, the present results revealed a double CF, for lay (regarding legal knowledge) individuals, but not for participants with more advanced levels of legal knowledge/experience with legal proceedings. As an empirical finding, this constitutes a salutary message regarding the ability of humans to embody rational decision making, in situations where there is a high expectation for such decision making. The double CF presents a challenge for decision models specifically developed to account for the CF and related fallacies. We focussed on one model, based on QPT. So far, QPT theory for the CF has been applied to the single CF, which is by far the most common finding. Modeling of the single CF with QPT involves incompatible questions, which lead to a psychological explanation based on how one question alters our perspective for the other. Regarding the double CF, we have outlined one possibility based on QPT, corresponding to compatible questions, and a “mixing” thought process; our outline was intended to simply show indicative calculations, noting that for a single CF only particular space structures will work. Psychologically this corresponds to a consideration of the two questions in a way that thoughts making each one individually more likely interfere with each other in the conjunctive case to produce probabilities inconsistent with CPT.

Generally, whether two questions are more likely to be represented as incompatible or compatible is currently approached as an empirical issue: there are simple empirical tests, such as order effects, which allow us to determine empirically whether two questions are compatible or incompatible. The QPT modeling indicates that for (legally) naïve participants consideration of one crime impacts on the consideration of the other crime. Perhaps for lay participants naïve familiarity with situations where a person is either generally guilty or not guilty at all influences their perception of how guilt for one crime affects guilt for another. Note, even though the QPT model is descriptive, it does accommodate results which are beyond any standard CTP approach. In order to develop the present intuitions into more inferential models, one challenge is to link various individual differences aspects of the participants (such as experience with particular situations) to key aspects of the modeling, notably compatibility vs. incompatibility. Notwithstanding these challenges for future work, the present results provide both additional evidence that legal decision making should be further scrutinized for potential fallacies and a theoretical framework with QPT which, however preliminary, offers some possible insights regarding human behavior.

## Ethics statement

This study was carried out in accordance with the recommendations of the Code of Ethics of the Polish Psychological Society with written informed consent from all subjects. The protocol was approved by the Ethics Committe at the Pedagogics and Psychology Faculty at the University of Silesia.

## Author contributions

BW and EP contributed to the conception and design of the study; BW organized the database; BW and EP performed the statistical analysis; BW and EP wrote the first draft of the manuscript.

### Conflict of interest statement

The authors declare that the research was conducted in the absence of any commercial or financial relationships that could be construed as a potential conflict of interest.
