# Corrigendum: Is There a Conjunction Fallacy in Legal Probabilistic Decision Making?

**DOI:** 10.3389/fpsyg.2018.02281

**Published:** 2018-11-27

**Authors:** Bartosz W. Wojciechowski, Emmanuel M. Pothos

**Affiliations:** ^1^Department of Clinical and Forensic Psychology, Institute of Psychology, University of Silesia of Katowice, Katowice, Poland; ^2^Department of Psychology, City, University of London, London, United Kingdom

**Keywords:** conjunction fallacy, legal decision making, quantum cognition, quantum probability theory, legal psychology

In the original article, there was an error.

In the Discussion section, a small error was made in one of the quantum computations, which requires minor adjustment of the discussion. None of the empirical results, analyses, and other conclusions are affected.

A correction has been made to *Discussion*, paragraph 7. The original sentence was: For a double CF, we have: *Prob*(A), *Prob*(B)>*Prob*(A&B), that is a CF occurs for both conjuncts. This has been corrected to: For a double CF, we have: *Prob*(A), *Prob*(B) < *Prob*(A&B), that is a CF occurs for both conjuncts.

A correction has been made to *Discussion*, paragraph 8. The original sentence was: For the participants with no legal background, we have a situation where *Prob*(A), *Prob*(B)>*Prob*(A&B), for when evaluating criminal cases for which the suspect was guilty for both crimes. This has been corrected to: For the participants with no legal background, we have a situation where *Prob*(A), *Prob*(B) < *Prob*(A&B), for when evaluating criminal cases for which the suspect was guilty for both crimes.

A correction has been made to *Discussion*, paragraph 10. The original sentences were: The observed results require an initial representation for the mental space in a tensor product structure as above, but also a thought process which “mixes” thoughts and beliefs between the two crimes (Pothos and Busemeyer, 2009; Broekaert et al., 2017). It is not our purpose presently to outline in detail a full cognitive model for the consideration of criminal cases and we focus on the technical elements of QPT that allow for coverage of the results (for more relevant details see e.g., Pothos and Busemeyer, 2009; Trueblood and Busemeyer, 2011; Pothos et al., 2013; Wang et al., 2013; Narens, 2014). This has been corrected to: The observed results motivate the consideration of an initial representation for the mental space in a tensor product structure as above, but also a thought process which “mixes” thoughts and beliefs between the two crimes (Pothos and Busemeyer, 2009; Broekaert et al., 2017). It is not our purpose presently to outline in detail a full cognitive model for the consideration of criminal cases and we focus on the technical elements of QPT potentially relevant for coverage of the results (for more relevant details see Pothos and Busemeyer, 2009; Trueblood and Busemeyer, 2011; Pothos et al., 2013; Wang et al., 2013; Narens, 2014).

A correction has been made to *Discussion*, paragraph 11, |a−c|^2^ has been replaced by |a|^2^+|c|^2^, and |*a*−*b*|^2^ has been replaced by |a|^2^+|b|^2^. A sentence has also been added, the corrected paragraph appears below.

$$\begin{array}{l} Prob\left(\text{​}Crime1_{yes};\psi \text{​}\right)=\text{​}P_{Crime1yes}⊗\;I\cdot U\cdot \psi^{2}\\ \;=\text{ ​}|\text{​}\left(\begin{array}{llll} 1 & 0 & 0 & 0\\ 0 & 1 & 0 & 0\\ 0 & 0 & 0 & 0\\ 0 & 0 & 0 & 0 \end{array}\right)\cdot \text{ ​}\left(\begin{array}{llll} 1 & 0 & 0 & 0\\ 0 & 0 & -1 & 0\\ 0 & -1 & 0 & 0\\ 0 & 0 & 0 & 1 \end{array}\right)\text{ ​}\cdot \text{ ​}\left(\begin{array}{l} a\\ b\\ c\\ d \end{array}\right)\text{ ​}{|}^{2}\text{​}=\text{​}|a{|}^{2}\text{​}+\text{​}|c{|}^{2} \end{array}$$

$$\begin{array}{l} \;Prob\left(Crime2_{yes};\psi \right)=|I⊗\;P_{Crime2yes}\cdot U\cdot \psi {|}^{2}=|a{|}^{2}+|b{|}^{2}\\ \;Prob(Crime1_{yes}\&Crime2_{yes};\psi )=|a{|}^{2} \end{array}$$

Recall that a mental state vector in QPT is normalized, therefore |*a*|^2^+|*b*|^2^+|*c*|^2^+|*d*|^2^ = 1. But it should be clear that this scheme still cannot accommodate a CF, which illustrates that only certain space structures can produce a single CF (e.g., as in Pothos and Busemeyer, 2009) and it is unclear whether a double CF is possible at all.

A correction has been made the *Discussion*, paragraph 12. A sentence was added, the corrected paragraphs appear below.

Overall, the present results revealed a double CF, for lay (regarding legal knowledge) individuals, but not for participants with more advanced levels of legal knowledge/experience with legal proceedings. As an empirical finding, this constitutes a salutary message regarding the ability of humans to embody rational decision making, in situations where there is a high expectation for such decision making. The double CF presents a challenge for decision models specifically developed to account for the CF and related fallacies. We focussed on one model, based on QPT. So far, QPT theory for the CF has been applied to the single CF, which is by far the most common finding. Modeling of the single CF with QPT involves incompatible questions, which lead to a psychological explanation based on how one question alters our perspective for the other. Regarding the double CF, we have outlined one possibility based on QPT, corresponding to compatible questions, and a “mixing” thought process; our outline was intended to simply show indicative calculations, noting that for a single CF only particular space structures will work.

Psychologically this corresponds to a consideration of the two questions in a way that thoughts making each one individually more likely interfere with each other in the conjunctive case to produce probabilities inconsistent with CPT.

The authors apologize for this error and state that this does not change the scientific conclusions of the article in any way. The original article has been updated.

## Conflict of interest statement

The authors declare that the research was conducted in the absence of any commercial or financial relationships that could be construed as a potential conflict of interest.
